# Identification of potential cell death-related biomarkers for diagnosis and treatment of osteoporosis

**DOI:** 10.1186/s12891-024-07349-6

**Published:** 2024-03-25

**Authors:** Mingliang Li, Xue Wang, Mingbo Guo, Wenlong Zhang, Taotao Li, Jinyang Zheng

**Affiliations:** 1 Department of Joint and Sports Medicine, Weifang Sunshine Union Hospital, Weifang, Shandong Province 261000 China; 2 Department of endocrinology, Weifang Sunshine Union Hospital, Weifang, Shandong Province 261000 China; 3 Department of spine 1, Weifang Sunshine Union Hospital, No. 9000, Yingqian Street, High-tech Zone, Weifang, Shandong Province 261000 China

**Keywords:** Osteoporosis, Programmed cell death-related genes, Diagnostic biomarkers, Immune cell infiltration, Machine learning

## Abstract

**Background:**

This study aimed to identify potential biomarkers for the diagnosis and treatment of osteoporosis (OP).

**Methods:**

Data sets were downloaded from the Gene Expression Omnibus database, and differentially programmed cell death-related genes were screened. Functional analyses were performed to predict the biological processes associated with these genes. Least absolute shrinkage and selection operator (LASSO), support vector machine (SVM), and random forest (RF) machine learning algorithms were used to screen for characteristic genes, and receiver operating characteristics were used to evaluate the diagnosis of disease characteristic gene values. Gene set enrichment analysis (GSEA) and single-sample GSEA were conducted to analyze the correlation between characteristic genes and immune infiltrates. Cytoscape and the Drug Gene Interaction Database (DGIdb) were used to construct the mitochondrial RNA-mRNA-transcription factor network and explore small-molecule drugs. Reverse transcription real-time quantitative PCR (RT-qPCR) analysis was performed to evaluate the expression of biomarker genes in clinical samples.

**Results:**

In total, 25 differential cell death genes were identified. Among these, two genes were screened using the LASSO, SVM, and RF algorithms as characteristic genes, including *BRSK2* and *VPS35*. In GSE56815, the area under the receiver operating characteristic curve of *BRSK2* was 0.761 and that of *VPS35* was 0.789. In addition, immune cell infiltration analysis showed that *BRSK2* positively correlated with CD56dim natural killer cells and negatively correlated with central memory CD4 + T cells. Based on the data from DGIdb, hesperadin was associated with *BRSK2*, and melagatran was associated with *VPS35*. *BRSK2* and *VPS35* were expectably upregulated in OP group compared with controls (all p < 0.05).

**Conclusions:**

BRSK2 and VPS35 may be important diagnostic biomarkers of OP.

**Supplementary Information:**

The online version contains supplementary material available at 10.1186/s12891-024-07349-6.

## Background

Osteoporosis (OP) is a bone disease characterized by bone tissue degeneration and reduced bone mass [1]. OP has high morbidity worldwide, especially among older adults [2]. Owing to increased bone fragility, approximately 9.0 million fractures occur annually [3], which significantly increases the burden on families and the quality of life. Senile and postmenopausal OP are the two primary types of OP. Traditionally, postmenopausal OP is characterized by excessive bone absorption [4], and senile OP is associated with a low bone turnover state, leading to impaired bone formation [5]. In clinical settings, OP is asymptomatic during the early stages. Typically, this disease is not diagnosed until bone fragility occurs. Thus, the early diagnosis of OP remains challenging. Currently, drug therapy with bisphosphonates, calcitonin, and fluorides is the primary treatment strategy for OP. The current treatment strategy is helpful for improving clinical symptoms and bone loss [6, 7]. However, the long-term clinical efficacy of these drugs remains limited. Moreover, owing to drug side effects and poor drug tolerance [8], a safer and more effective treatment strategy is urgently required.

In tissues, cell apoptosis acts as a defense mechanism to prevent non-functional cell accumulation; however, if mutations occur and cell apoptosis cannot be activated, excessive cell death or evasion from apoptosis may cause the development of various diseases [9]. Programmed cell death is widely involved in skeletal repair, maintenance, and development, and is closely related to the pathogenesis of osteoarthritis and OP [10]. Programmed cell death-related genes (PCD) have been extensively explored in many diseases. Different gene families, such as caspases, the p53 gene, the superfamily of tumor necrosis factor receptors, and the B cell lymphoma-2 family of genes, are associated with apoptotic processes [9]. During bone metabolism regulation, PCDs play critical roles by modulating bone cell activity; hence, molecules targeting programmed cell death may be valuable biomarkers for OP development. Various bone cell functions contribute to bone formation and bone resorption [11]. When cells cannot meet these requirements, the process of bone resorption and formation is disrupted. Consequently, bone remodeling may fail and OP may occur. In OP, chondrocyte apoptosis is accepted as an important step in endochondral ossification. The role of PCDs has been explored previously. For example, in OP, upregulated BCLXL inhibits osteoblast apoptosis and maintains normal bone structure [12]. Therefore, we believe that PCDs may participate in the pathogenesis of OP and may serve as biomarkers for OP treatment and diagnosis.

To explore the potential roles of PCDs in OP, the GSE56815 and GSE7158 microarray datasets were downloaded from Gene Expression Omnibus (GEO) as training and validation datasets, respectively. Differentially expressed PCDs were screened and functional analysis was performed to predict the biological processes enriched by these genes. After screening, a receiver operating characteristic (ROC) curve was used to evaluate the diagnostic value of the characteristic genes. Furthermore, the correlation between the characteristic genes and immune infiltrates was explored. The mitochondrial RNA (miRNA)-mRNA-transcription factor (TF) network was constructed, and small-molecule drugs related to the characteristic genes were explored. Thus, BRSK2 and VPS35 may be important diagnostic and treatment biomarkers for OP.

## Methods

### Data resource

Two microarray datasets (accession numbers: GSE56815 and GSE7158) were downloaded from the GEO database (https://www.ncbi.nlm.nih.gov/). GSE56815 included circulating monocytes from 40 subjects each with extremely high BMD (bone mineral density) and 40 subjects with extremely low BMD according to the hip Z-score. The dataset was used as the training set and analyzed based on the GPL96 [HG-U133A] Affinometrix Human Genome U133A array annotation platform. Peak bone mass (PBM) is another important determinant of osteoporosis [13]. GSE7158, including circulating monocytes from 14 subjects with extremely high Peak bone mass (PBM) and 12 subjects with low PBM, were enrolled as validation data, which were analyzed based on the GPL570 [HG-U133-Plus_2] Affimatrix Human Genome U133 Plus 2.0 Array annotation platform files.

### Identification of differentially expressed genes (DEG)

Before DEG analysis, we performed Principle Component Analysis at gene expression level to evaluate the data quality. As shown in Supplementary Fig. 1, samples in GSE56815 were assigned into two groups separately and there was no outlier sample. Thus, the dataset could be used for DEG analysis. The OP related DEGs between high and low BMD groups in GSE56815 were analyzed using the Limma package [14] in R. Benjamin, and the Hochberg method [15] was used for multiple test correction. DEGs were visualized using Volcano map and heatmap using R packages “ggplot2” and “pheatmap” in R. The cutoff values were defined as an adjusted P-value < 0.05.

### Weighted gene co-expression network analysis (WGCNA)

WGCNA is a valuable tool for identifying modules of highly correlated genes by clustering genes with similar expression levels, and can analyze the correlation between modules and specific traits or phenotypes [16]. First, the samples were clustered to detect the outliers. The weight value was calculated based on the Picksoft Threshold function of WGCNA. A thresholding power β value of 6 (scale-free R2 = 0.85) was determined for constructing a scale-free network. Gene co-expression network was constructed and modules were clustered based on dynamic tree cutting algorithm with a minimum size of 70 genes. MEDissThres was set as 0.2 to combine the similar modules. The relationship between modules and clinical traits were analyzed by Pearson’s correlation coefficient. Modules with a correlation of P value < 0.05 and a correlation coefficient ≥ 0.3 were selected as key modules, and genes within more than one key module were defined as hub genes.

### Selection of differentially expressed PCDs and function enrichment analysis

Twelve types of PCDs were obtained from Zou et al. [17], and 1254 PCDs were obtained after deduplication (Supplementary file 1). The online tool jvenn (http://jvenn.toulouse.sra.fr/app/example. html) was used to detect the intersection of DEGs, PCDs, and hub genes as differentially expressed PCDs. To understand the biological role of the intersecting genes, Gene Ontology (GO) enrichment analysis was performed using R package “cluster Profiler” [18] and the results were visualized by R package “ggplot2” [19]. Genes of interest were annotated in three categories of biological process (BP), molecular functions (MF), and cellular components (CC) [20]. A P value < 0.05 was defined as the significance threshold.

### Protein-protein interaction (PPI) network construction

Given that PPI networks are involved in various stages of processes such as biological signal transduction, gene expression regulation, and cell cycle regulation [21], PPI network is crucial for the investigation of disease occurrence and development. STRING database (http://stringdb.org/) [22] was employed to generate a PPI network of differentially expressed PCDs. The protein pairs with confidence level of 0.4 were collected for further analysis. In the present study, PPI network was visualized using Cytoscape [23], in which the nodes represented proteins and edges indicated protein-protein interaction. The CytoNCA plugin was used to analyze the connectivity of PPI network.

### Machine learning methods for screening biomarkers

The biomarker genes were further filtered by three machine learning methods, including Least absolute shrinkage and selection operator (LASSO), Support Vector Machines (SVM) and Random forest (RF).

LASSO regression is a contraction estimation method that minimizes the sum of squared residuals under constraints [24]. Based on the expression values of the differentially expressed PCDs and the grouping information of the samples in GSE56815, a LASSO regression prediction sample classification was constructed. To reduce the feature dimension, parameters of the R software “glmnet” package (version 4.0–2) were set as: family="binary,” type. measure="class,” nfold = 10.

SVM is a supervised learning method that uses a training dataset to build a classification model and a validation dataset to validate the model [25]. The intersecting genes were sorted using linear and non-linear SVMs using the “e1071” R package. Recursive feature elimination was used to analyze the importance and ranking of each gene as well as the error rate and accuracy of each iterative combination [26]. The lowest error rate was selected as the optimal combination and the corresponding gene was defined as the characteristic gene. RF is a composite supervised learning method, which is also an extension of decision trees [27]. The RF method in the R package was used to analyze the intersecting genes for RF analysis to screen for characteristic genes. The genes obtained from the RF algorithm were ranked by “Mean DecreaseAccuracy” and “Mean DecreaseGini” respectively. Finally, characteristic genes screened using LASSO, SVM, and RF were intersected using the jVenn tool.

### Diagnostic value analysis and differential expression of biomarkers

To evaluate the diagnostic value of each biomarker, ROC curves based on differentially expressed PCDs were constructed in the dataset GSE56815. Briefly, the gene expression value in each sample was extracted. R package “pROC” was utilized to conduct ROC curve analysis by taking samples grouping as outcome variable and the gene expression value as the independent variable. Furthermore, ROC curves based on selected biomarkers in GSE7158 were also constructed to verify the diagnostic value of selected genes. Subsequently, the expression levels of differentially expressed PCDs from the GSE56815 dataset and GSE7158 validation set were extracted, and the expression levels of the biomarkers were compared between OP and controls using the wilcox.test method in R package “ggplot2 [19].”

### Construction of column line diagram model

In order to further verify the diagnostic ability of differentially expressed PCDs, the “RMS” package was used to build the nomogram model of biomarkers based on the sample grouping information and the expression of biomarkers. Furthermore, the predictive value of the column chart model was evaluated by drawing a calibration curve using the calibrate function in the “RMS” package, and the column chart model ROC curve was drawn using the “pROC” package [28].

### Gene set enrichment analysis (GSEA)

The enriched pathways of the selected genes were analyzed using GSEA software (V4.0.3) based on the expression values of the two biomarkers. The parameters were set as follows: gene set database, c2.cp.kegg.v7.5.1. symbols.gmt; gene sets database, c5.go.bp.v7.5.1.symbols.gmt. Pearson correlation coefficients for each gene and biomarker were calculated, and the correlation coefficients between each biomarker and the other genes were sorted in descending order (metric for ranking genes: Pearson, gene list sorting mode: real, gene list ordering mode: descending). The above gene set was set as the background gene, and the pathways enriched by these genes with |NES| > 1 and NOM P value < 0.05 were defined as enriched pathways.

### Immune cell infiltration analysis

The infiltration abundance of 28 immune cells in all samples in the training set was analyzed using the R package “GSVA” [29] and single-sample GSEA algorithm [30]. The Spearman correlation between all immune cells and differentially expressed PCDs was calculated using the R package “coreplot.” Immune cells significantly related with OP were screened by drawing a box plot using the wilcox.test method in R package “ggplot2 [19].”

### Construction of miRNA-mRNA-TF network

The MiRwalk database contains the predicted and experimentally validated miRNA-gene interaction pairs [31]. In the present study, we used miRwalk3.0 database to screen experimentally validated miRNA-biomarker interactions based on the 3’ untranslated region binding site position, and the threshold was set as binding probability ≥ 0.95. The NetworkAnalyst database was used to predict the TF for the selected biomarkers. The parameters were set as follows: Specify organization, H. sapiens; set ID type, official gene symbol; and TF gene interaction database, ENCODE. Finally, the miRNA-mRNA-TF regulatory network was constructed using Cytoscape software [32]. Finally, small-molecule drugs related to the biomarkers were selected using the DGIdb [33](https://dgidb.genome.wustl.edu/).

### Reverse transcription real-time quantitative PCR (RT-qPCR) analysis

Ten subjects aged 45–55 years old were recruited from the Weifang Sunshine Union Hospital. The hip BMD was measured by a dual-energy X-ray bone densitometer. Then, BMD was transformed into a Z score as previous description [34, 35]. Subjects were assigned into high BMD (control, n = 10) and low BMD (OP, n = 10) group. The whole blood samples were collected from all the included individuals with informed consent. Ethics Committee of our hospital approved this study and all the procedures were conducted complied with the rules and guidelines of Declaration of Helsinki.

Peripheral blood monocytes (PBMs) were isolated from whole blood samples using the commercial monocyte isolation kit (Miltenyi Biotec Inc, Auburn, CA). Total RNA extraction was performed with the application of Trizol reagent. cDNA was prepared using the SuperScript VILO cDNA synthesis kit (Invitrogen) per the manufacturer’s protocols. Genes were amplified under the Applied Biosystems 7000 sequence detection system, following the conditions of 30 s at 95℃, 45 cycles of 95℃ for 10s and 60℃ for 30s. GAPDH was used as the reference gene. The PCR primers were summarized in Table 1.

**Table 1:** Primer sequence for RT-qPCR

Gene symbol	Forward	Reverse
BRSK2	5’-ACATCCGCATCGCAGACTTT-3’	5’-CGCAAGTTGTCATCGTCGAAG-3’
VPS35	5′-CCGCTCGAGATGCCTACAACACAGCAGTCCC-3′	5′-CGGGAATTCTTAAAGGATGAGACCTTCATAAATT-3′
GAPDH	5’-ATGGGGAAGGTGAAGGT-3’	5’-AAGCTTCCCGTTCTCAG-3’

## Results

### DEGs selection

**Fig. 1:**
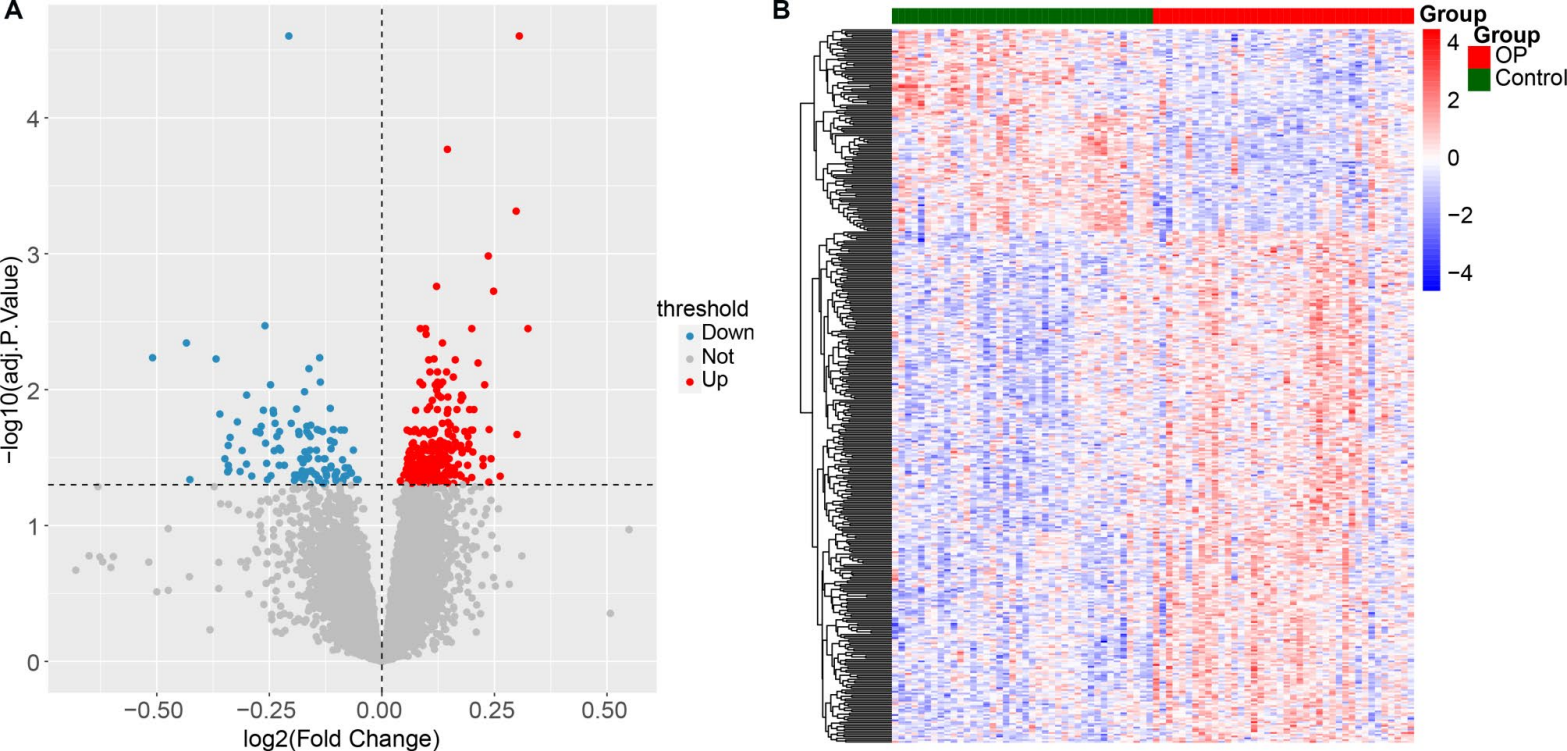
Volcano plot and heatmap of differentially expressed genes (DEGs). **A**: Volcano plot of DEGs; Horizontal axis represents difference multiple, and vertical axis represents - log10 (adj. P. Value). Each point in the graph represents a gene, and the blue and red dots represent DEGs. The red dots indicate upregulated genes, the blue dots indicate downregulated genes, and the gray dots indicate genes with non-significant difference. **B**: Each small square represents each gene, and its color represents the expression level of the gene. The darker the color represents the higher expression level. Each row represents the expression level of each gene in different samples, and each column represents the expression level of all differential genes in each sample. The left tree graph represents the clustering analysis results of different genes from different samples

In this study, a total of 399 DEGs were identified as OP related genes (Supplementary file 2). Compared with control group, 286 genes were upregulated and 113 were downregulated in OP group (Fig. 1A). Samples were clearly distinguished based on their differential expression profiles (Fig. 1B).

### WGCNA network construction

**Fig. 2:**
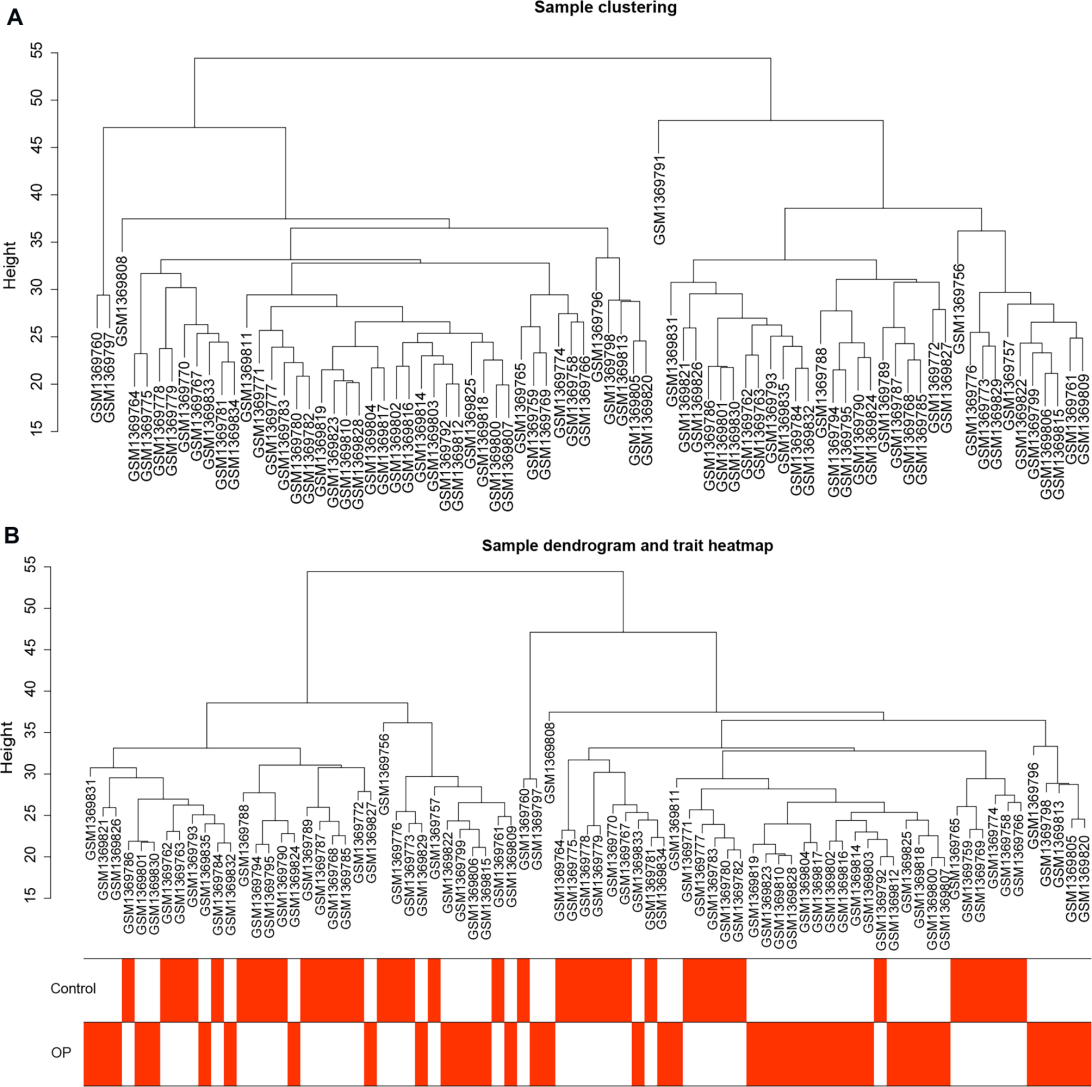
Cluster analysis of dataset samples, data Sample Clustering, and Phenotypic Information. **A**: cluster analysis of dataset samples; the branches in the figure represent samples, and the ordinate represents the height of hierarchical clustering. **B**: Data Sample Clustering and Phenotypic Information; the upper half of the figure shows the clustering situation, the lower half shows the phenotype, and the red represents the corresponding phenotypic traits

**Fig. 3:**
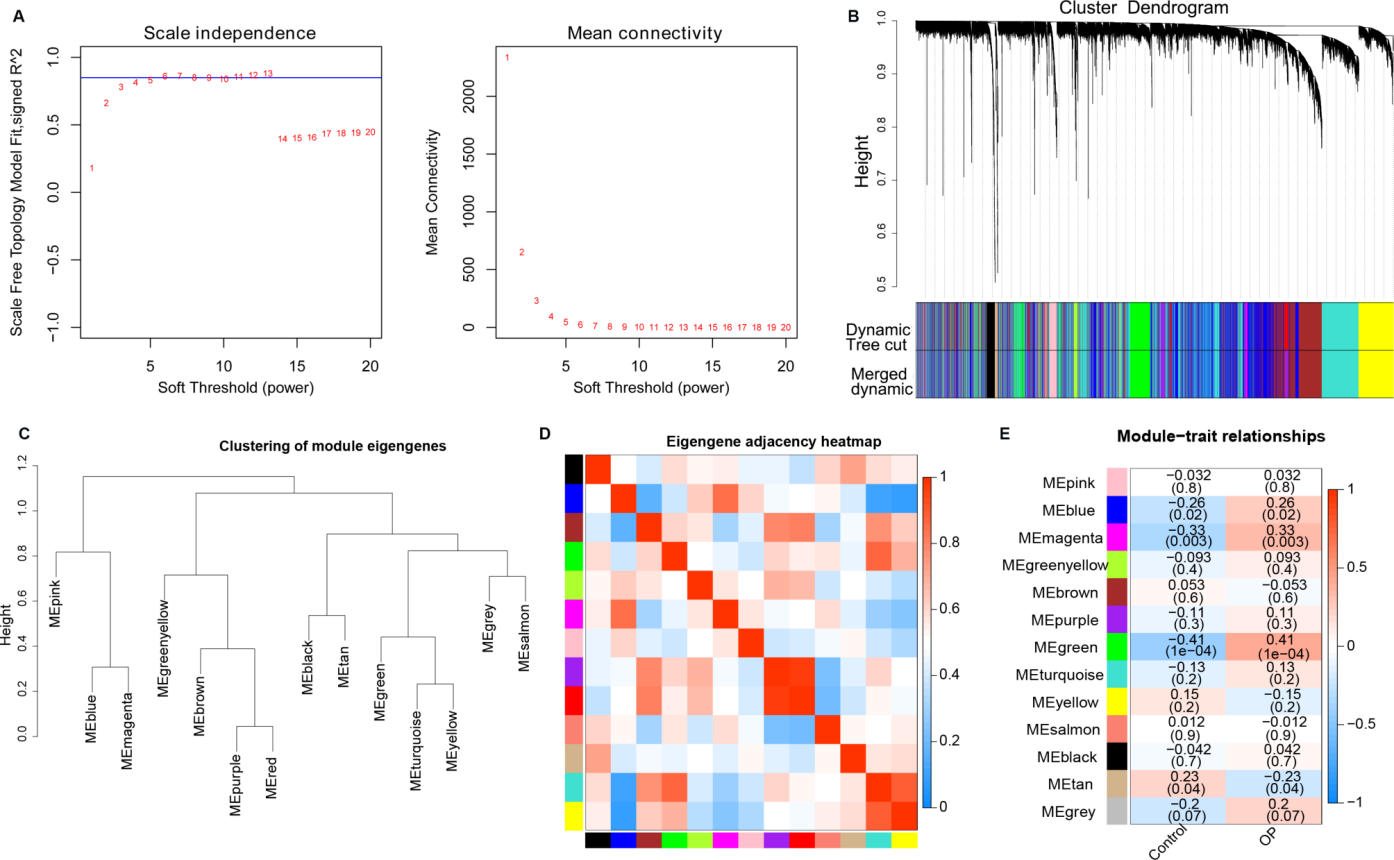
The results of weighted gene co-expression network analysis (WGCNA). **A**: Scale free soft threshold distribution; The horizontal axis represents the power value of the weight parameter. The vertical axis of the left graph represents Scale Free Topology Model Fit, that is, signed R^2. The higher the square of the correlation coefficient represents the closer the network approaches the scale-free distribution. The vertical axis of the right graph represents the mean value of all gene adjacency functions in the corresponding gene module. **B**: Module Clustering Tree; the module clustering tree graph gene is divided into various modules through hierarchical clustering, and different colors represent different modules. **C**: Clustering of module eigengenes. **D**: Clustering of module heatmap. **E**: Heatmap of the module-clinical traits relationships

Sample cluster analysis was first conducted to investigate the difference in samples in GSE56815 dataset. The sample clustering dendrograms shows that GSM1369791 sample is different from others (Fig. 2A). After removing the outlier sample GSM1369791, the association between the modules and the clinical characteristics of the samples was analyzed. All DEGs were grouped into modules based on similar expression patterns (Fig. 2B). A soft threshold of six was selected to satisfy the scale-free topology with R^2^ = 0.85 (Fig. 3A).

### Key module identification and hub genes collection

Subsequently, a MEDissThres of 0.2 was used to merge similar modules and 13 modules were obtained (Fig. 3B). The characteristics of the samples were sorted and added to the clustering graph to construct a sample clustering and clinical trait heatmap (Fig. 3C). The correlation between different modules is shown in Fig. 3D. As shown in ​Fig. 3E, MEgreen (R^2^ = 0.41, P = 1e-04) showed the highest negative association with the Gleason score, whereas MEmagenta (R^2^ = 0.33, P = 0.003) showed the highest positive correlation with the Gleason score. Thus, the green module containing 911 genes and magenta module containing 138 genes were the most significant modules. The Supplementary file 3 shows the correlations of genes in significant modules. Finally, a total of 1049 hub genes were collected for further analysis.

### Differentially expressed PCDs, functional analysis, and PPI network

**Fig. 4:**
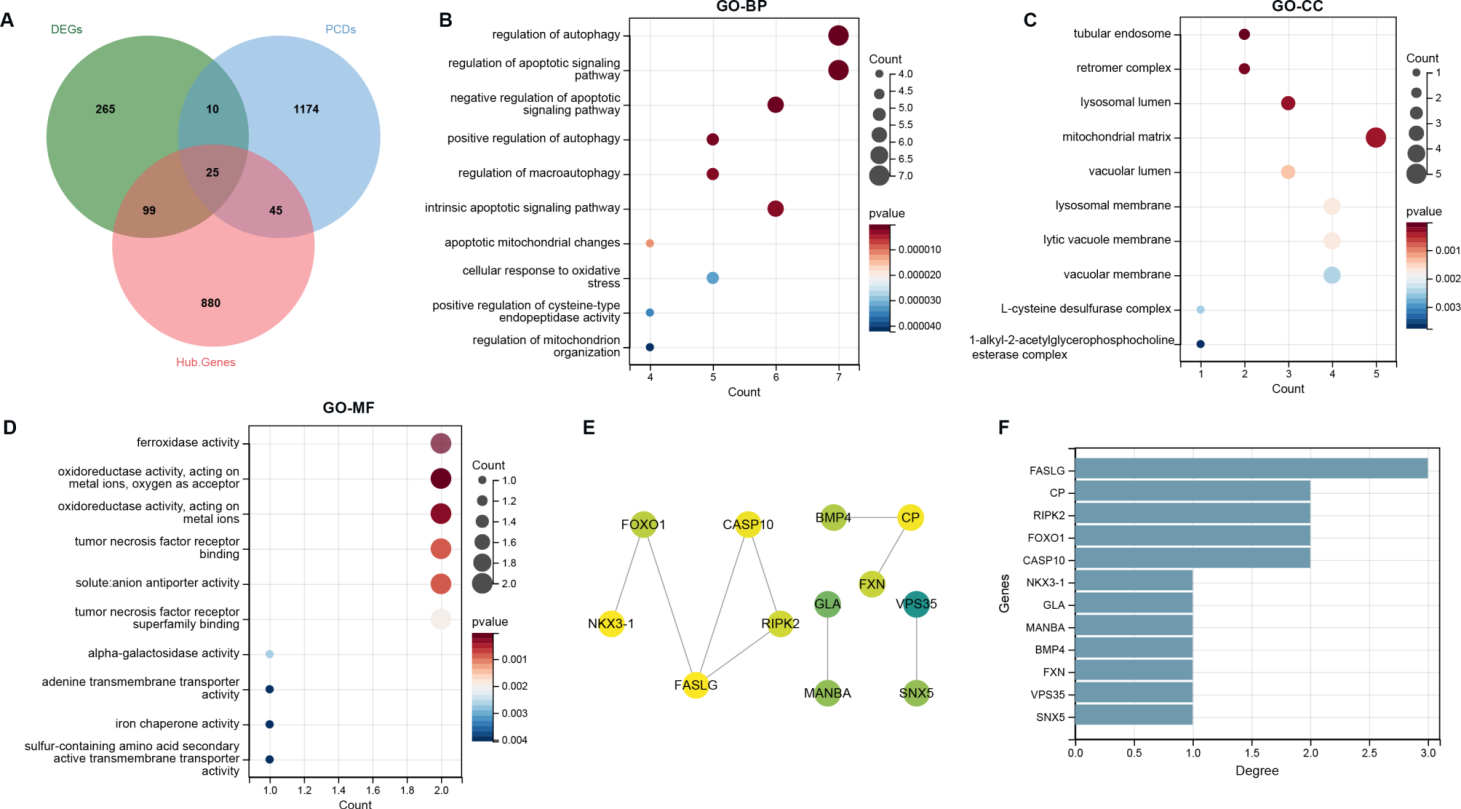
Immune-related programmed cell death genes (PCDs) and functional analysis. **A**: Venn diagram for selecting Immune-related PCDs; B: Bubble Chart of TOP10 GO biological process enrichment; **B**: Bubble Chart of TOP10 GO cellular component enrichment; **C**: Bubble Chart of TOP10 GO molecular function enrichment; The vertical axis represents the enriched GO descriptions, the horizontal axis represents the number of genes enriched by the pathway, the color represents the p value. The size of the bubble represents the number of intersecting genes contained in the description, and the larger the bubble represents the more genes it contains; **D**: diagram of protein-protein interaction network; **E**: Bar chart of PPI Network node connectivity

Total 25 differentially expressed PCDs were obtained by intersecting 399 DEGs, 1049 hub genes and 1254 PCDs (Fig. 4A), which were used for GO analysis. The top 10 significant GO terms ranked by p values were visualized. The results showed that these genes were significantly enriched in the regulation of autophagy, regulation of the apoptotic signaling pathway, and negative regulation of the apoptotic signaling pathway related GO BP (Fig. 4B). For CC, the differentially expressed PCDs were mainly enriched in tubular endosome, retromer complex, and lysosomal lumen (Fig. 4C) and in MF category, the genes were closely associated with ferroxidase activity, oxidoreductase activity, acting on metal ions, and oxygen as acceptors (Fig. 4D). As shown in Fig. 4E, a PPI network of the 25 PCDs was constructed comprising nine edges connecting 12 nodes. In the PPI network, FASLG exhibited the highest connectivity, followed by CP, RIPK2, FOXO1, and CASP10 (Fig. 4F).

### Screening of OP signature genes by machine learning analysis

**Fig. 5:**
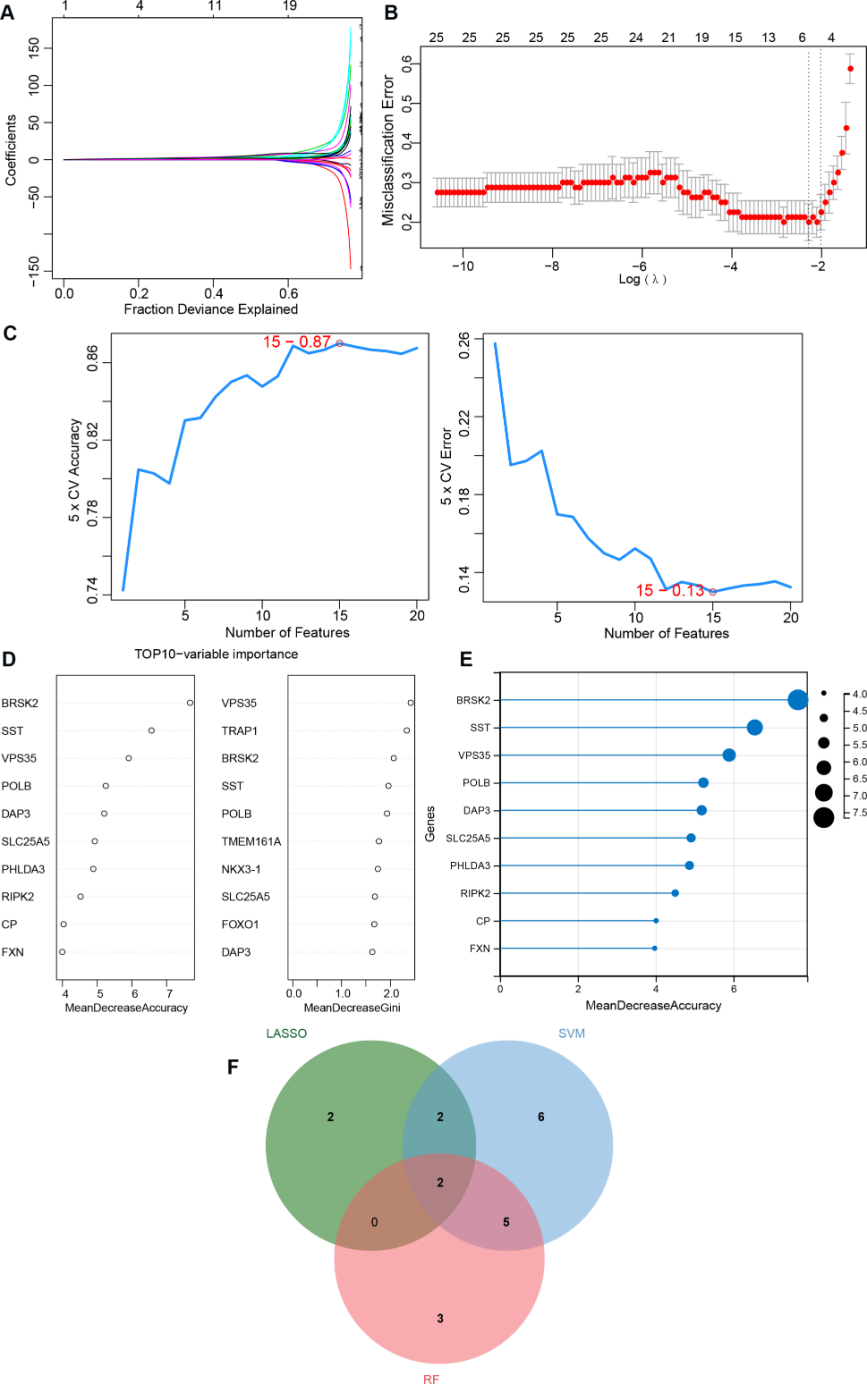
Machine learning for screening biomarkers. **A**: LASSO Logic Coefficient Penalty Graph; Each curve represents the variation trajectory of each independent variable coefficient, the y-axis represents the coefficient value, and the upper x-axis represents the number of non-zero coefficients in the model; **B**: LASSO Logic Coefficient Penalty Graph; The horizontal axis represents log (Lambda), while the vertical axis represents the error of cross validation; **C**: Support Vector Machine Model Accuracy (Left) and Error Rate (Right); **D**: Top 10-variable importance. “mean Decrease accuracy “indicates the degree of reduction in the accuracy of random forest prediction. The higher the value represents the greater the importance of the variable; “mean decrease Gini “calculates the impact of each variable on the heterogeneity of observations at each node of the classification tree. The higher the value represents the greater importance of variables; **E**: Lollipop map of top10 characteristic gene; **F**: Venn diagram for selecting biomarkers

The OP-associated biomarkers were further filtered by three machine learning methods. Based on the LASSO model, graphs of gene coefficients and error plots of cross-validation were constructed (Fig. 5A and B). Six characteristic genes were screened: *BRSK2, NKX3-1, SLC7A11, SNX5, TMEM161A*, and *VPS35*. To verify the accuracy of the SVM model under different features, we used a 5-fold cross validation to calculate the accuracy of the different feature combination models. The model achieved the highest accuracy after incorporating the first 15 features (Fig. 5C). Fifteen feature genes were identified. The RF results showed an order according to the mean decrease accuracy of the enrolled genes (Fig. 5D). The top ten genes included *BRSK2, SST, VPS35, POLB, DAP3, SLC25A5, PHLDA3, RIPK2, CP*, and *FXN* (Fig. 5E). Finally, two genes (*BRSK2* and *VPS35*) were screened by intersecting the LASSO, SVM, and RF results (Fig. 5F) and were used for further analysis.

### Diagnostic value of BRSK2 and VPS35 in OP

**Fig. 6:**
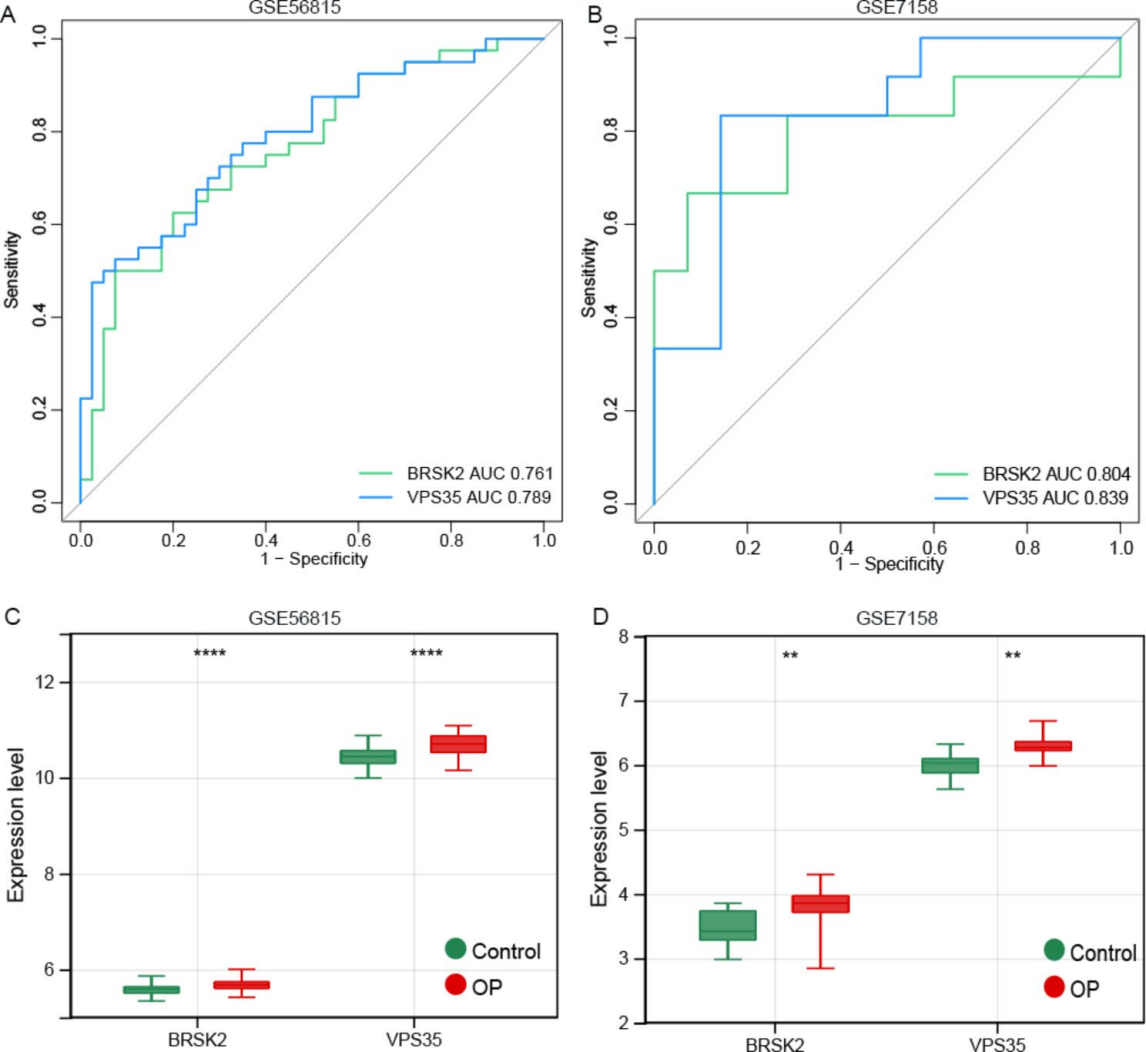
Diagnosis value of BRSK2 and VPS35 in osteoporosis. **A**: Receiver operating characteristic (ROC) curve of BRSK2 and VPS35 in dataset GSE56815; **B**: ROC curve of BRSK2 and VPS35 in GSE7158; **C**: Expression box diagram of BRSK2 and VPS35 in dataset GSE56815; **D**: Expression box diagram of BRSK2 and VPS35 in dataset GSE7158. Versus control, *p < 0.05, **p < 0.01, *** p < 0.001, **** p < 0.0001

In GSE56815 cohort, the area under the receiver operating characteristic curve (AUC) of *BRSK2* was 0.761 and that of *VPS35* was 0.789, suggesting that both *BRSK2* and *VPS35* have the ability to distinguish between OP and healthy controls (Fig. 6A). In GSE7158, the AUC of the two genes were more than 0.8 (*BRSK2* AUC: 0.804; *VPS35* AUC: 0.804), verifying the valuable diagnostic ability (Fig. 6B). Figure 6C and D show upregulated *BRSK2* and *VPS35* in OP compared with that in the control in both GSE56815 and GSE7158 (P < 0.01).

### Nomograph model

**Fig. 7:**
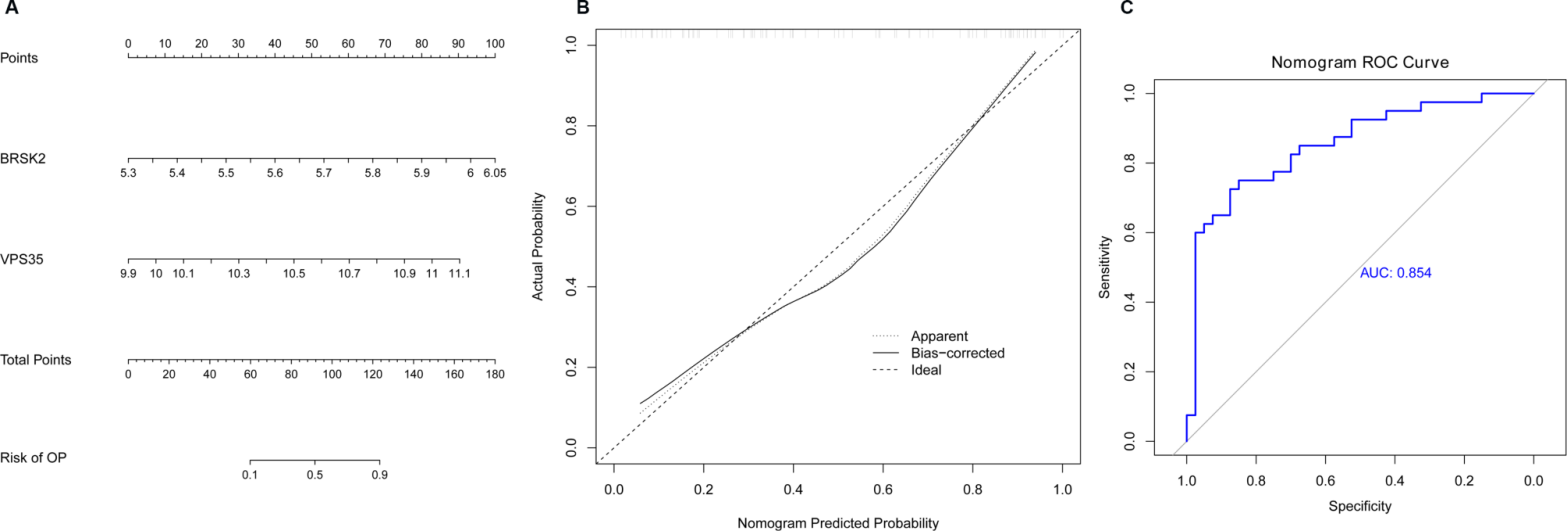
Construction of column line diagram model. **A**: Nomogram model of BRSK2 and VPS35; **B**: Calibration curve for evaluating the predictive ability of column chart models; **C**: Receiver operating characteristic curve of the column chart model

The nomograph model is constructed to verify the predictive power of the two biomarker genes (Fig. 7A). The calibration curve showed that the error between the actual disease risk and the predicted risk of the calibration curve was very small, indicating that the column chart model had high prediction accuracy for OP diseases (Fig. 7B). The ROC curve showed that the AUC value of the column chart model was greater than 0.85, indicating that the column chart model had the predictive ability to distinguish OP from normal samples (Fig. 7C).

#### Functional role of BRSK2 and VPS35 in OP

To gain the insight of the involvement of biomarker genes in OP, GSEA was employed to analyze OP related biological process and pathways. The results showed that *BRSK2* was mainly enriched in BPs, such as the apoptotic process involved in morphogenesis and microtubule organizing center localization, and Kyoto Encyclopedia of Genes and Genomes (KEGG) pathways such as ether lipid metabolism. *VPS35* was mainly enriched in BPs, such as regulation of glycoprotein metabolic processes and poly a plus mRNA export from the nucleus, and KEGG pathways, such as dilated cardiomyopathy and RNA degradation (Supplementary Fig. 2).

### Correlation of biomarkers and immune cells in OP

**Fig. 8:**
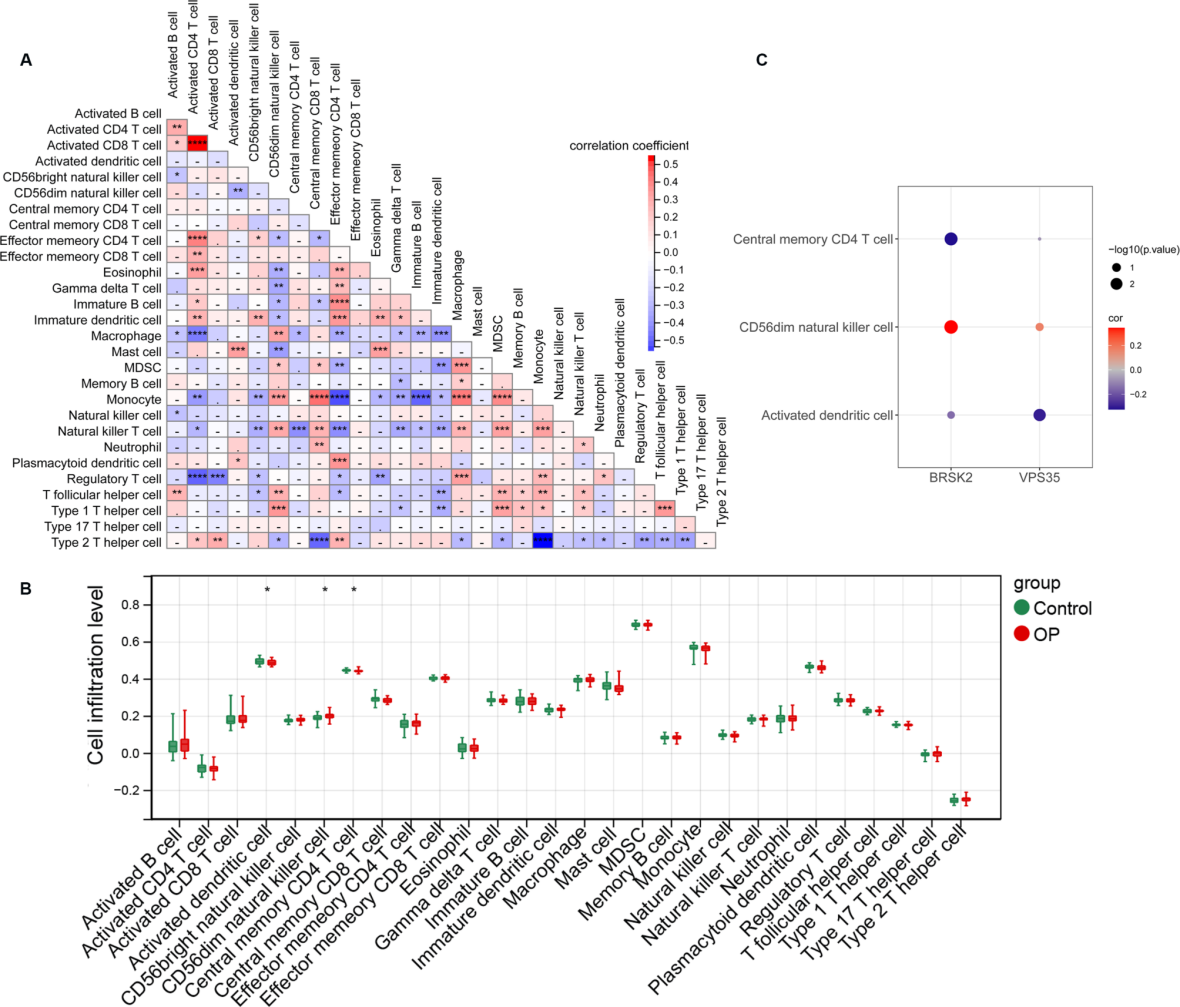
Correlation of biomarkers and immune cells in osteoporosis. **A**: Heatmap of correlation between immune cells; **B**: 28 immune cell expression box plots between sample groups; versus *p < 0.05, **p < 0.01, ***p < 0.001, ***p < 0.0001, -p > 0.05; **C**: Bubble chart of correlation between biomarkers and differential immune cells

The correlation between the biomarkers and immune cells was investigated and the heatmap of the correlation is shown in Fig. 8A. Significant differences were observed in three types of immune cells between OP and control groups, including activated dendritic cells, CD56dim natural killer cells, and central memory CD4 + T cells (Fig. 8B). A bubble chart of the correlation between biomarkers and differential immune cells shows that *BRSK2* is positively correlated with CD56dim natural killer cells and negatively correlated with central memory CD4 T cells, whereas *VPS35* is negatively correlated with activated dendritic cells (Fig. 8C).

#### The miRNA-mRNA-TF network and small molecule drugs

**Fig. 9:**
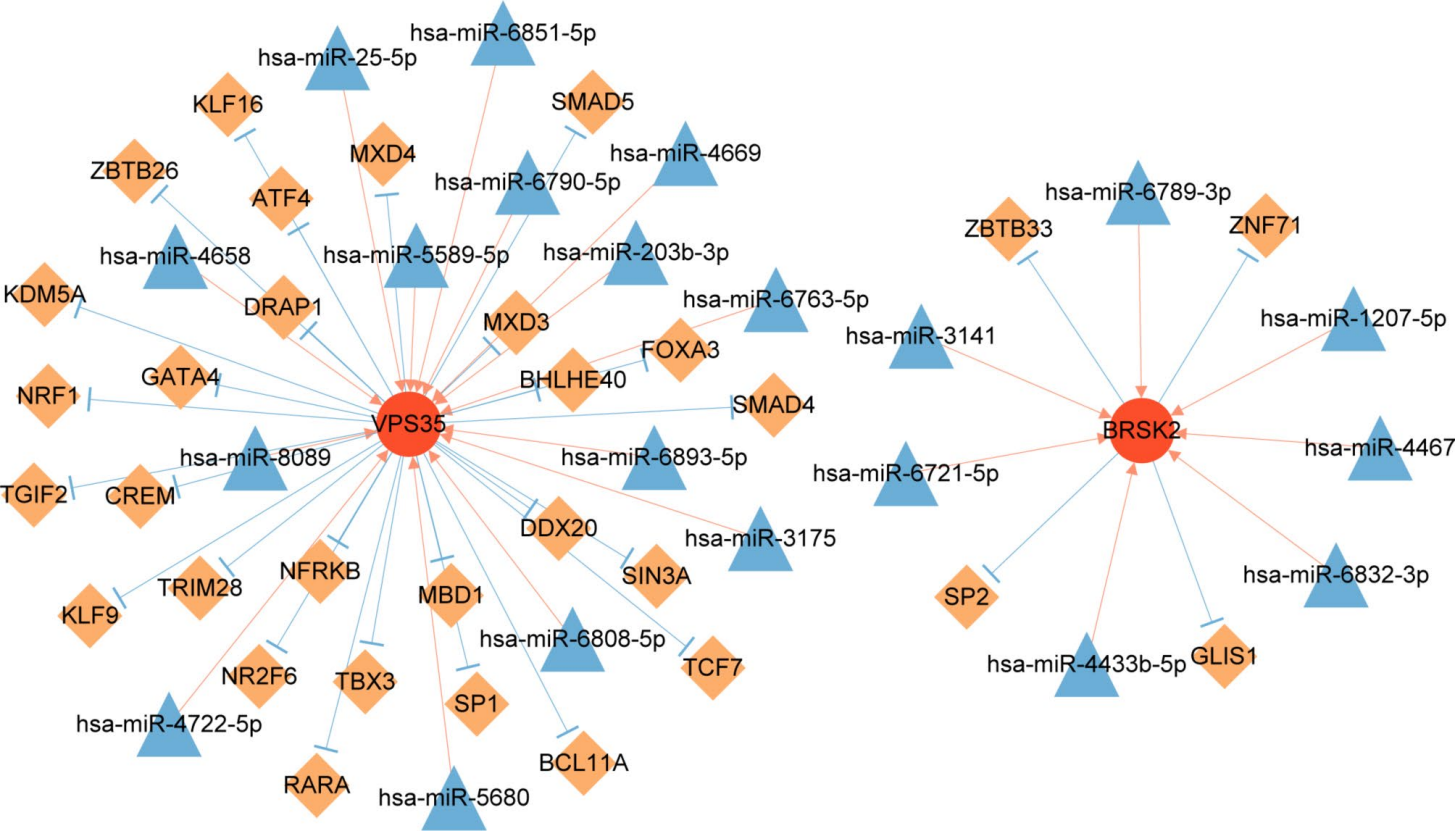
MiRNA-mRNA-transcription factor regulatory network.Red circle, biomarker gene; blue triangle, miRNA; orange square, transcription factor

MiRNAs and TFs play key roles in regulating gene expression and functions of gene encoding proteins. As shown in Fig. 9 and 406 interaction pairs were identified in the miRNA-mRNA-TF network, which included 21 miRNAs, two mRNAs, and 31 TFs. Based on the data from DGIdb, hesperadin was associated with *BRSK2*, and melagatran was associated with *VPS35*.

#### RT-qPCR confirmation

**Fig. 10:**
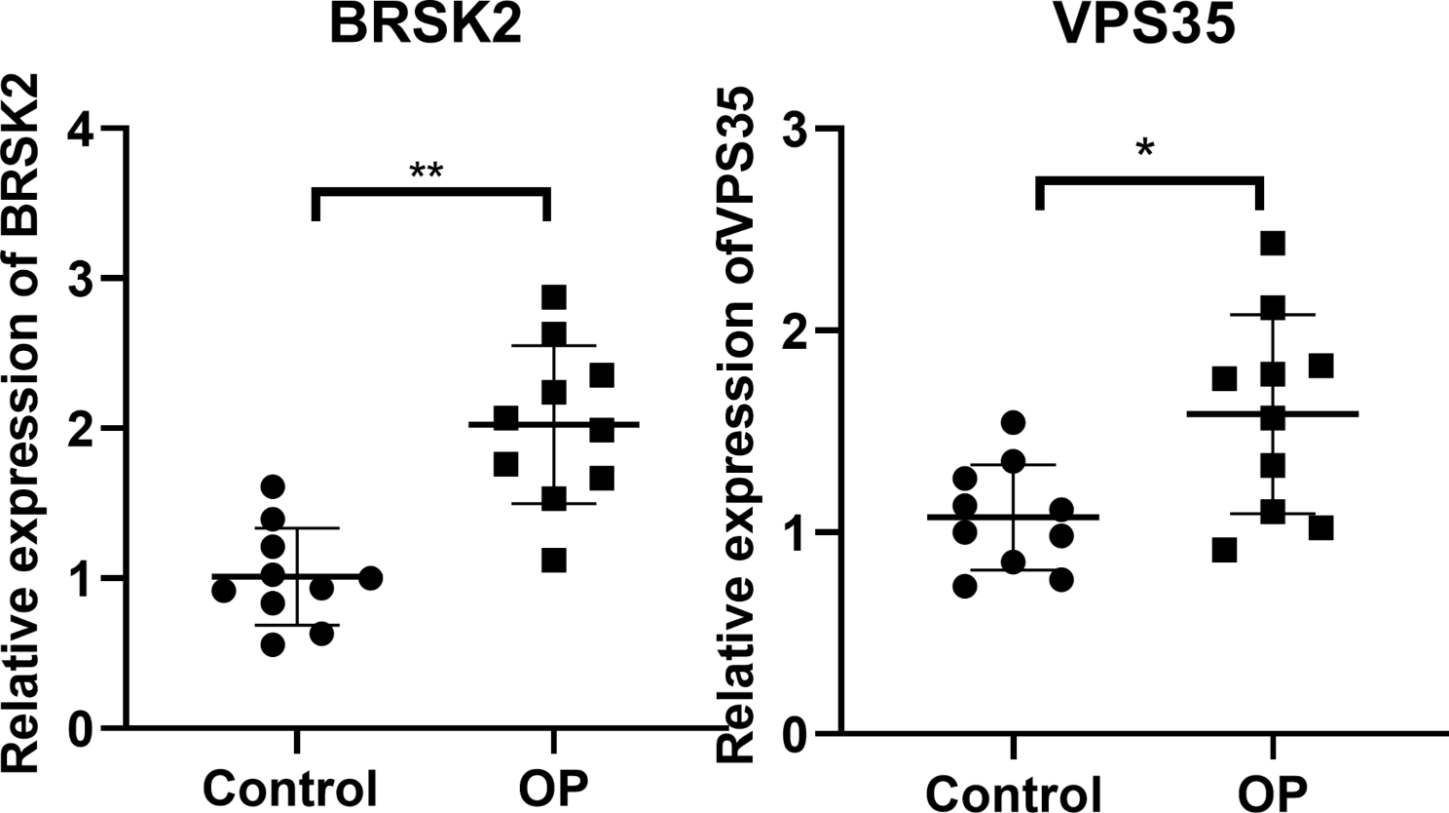
RT-qPCR validation of the biomarkers. Peripheral blood monocytes of ten subjects with high BMD and ten individuals with low BMD were collected for RT-qPCR assay. *p < 0.05 and **p < 0.01, compared with OP group with control group

To confirm the significantly differential expression of the biomarker genes in clinical samples, *RT-qPCR* analysis was performed. Consistent with the bioinformatics analysis in GSE56815 and GSE7158 datasets, *BRSK 2* and *VPS35* were expectably upregulated in OP patients compared with controls (all p < 0.05, Fig. 10).

## Discussion

OP is a complex bone disease characterized by deterioration of bone tissue microarchitecture and reduced bone mass. PCDs plays a critical role in the regulation of bone metabolism. Thus, we aimed to explore the valuable PCDs that contribute to OP development.

Here, *BRSK2* and *VPS35*, selected as the differentially expressed PCDs are vital for OP diagnosis. Programmed cell death is a cell death process that maintains homeostasis in the internal environment and is induced by a certain signal. The critical role of PCDs in regulating bone metabolism has been well demonstrated, and drugs targeting specific regulatory molecules in PCDs play valuable roles in preventing OP [11]. *VPS35* is critical for transmembrane proteins [36], is upregulated in osteoblasts and osteoclasts, and is activated by deregulating RANK signaling to prevent OP deficits [37]. The study by Raychaudhuri et al. demonstrated the roles of *VPS35* in osteoclast and osteoblast activity regulation in OP and put forward that *VPS35* may be a potential diagnostic biomarker of osteoporosis [38]. A similar conclusion was supported by Xia et al. based on data from GSE56815 and GSE56814 [39]. In the current study, we also identified a potential diagnostic role for *VPS35*.

In the present study, *BRSK2* was selected as a critical biomarker of OP. It is well known that *BRSK2* is important in cell cycle regulation, neuronal and axonogenic polarization, and insulin secretion. Although few studies have demonstrated the regulatory role of *BRSK2* in OP, the function of senescent and differentiated cells in age-related pathologies, including OP, has been well reported [40]. In addition, we showed that the level of *BRSK2* positively correlated with CD56dim natural killer cells and negatively correlated with central memory CD4 + T cells in OP. Natural killer cells are innate immune cells that act as the first responders to immunological processes [41, 42] and have been accepted as important regulators of senescent cell immune surveillance [43].

Several studies have demonstrated the association between the number and distribution of natural killer cell subsets and age-related diseases [44]. Collectively, the data in the present study showed a valuable diagnostic role for *BRSK2*, and the levels of this gene were related to the number of CD56dim natural killer cells, suggesting that *BRSK2* may be important for the OP process.

Based on data from DGIdb, hesperadin and melagatran were selected as potential treatment drugs for OP by targeting *BRSK2* and *VPS35*, respectively. Hesperadin is an inhibitor of human Aurora kinase, which is widely used to inhibit tumor growth and ameliorate cardiac reperfusion injury [45]. Moreover, the inhibitory role of melagatran in osteoblasts and its pronounced influence on cellular metabolism has been demonstrated [46]. Taken together, the treatment efficiency of hesperadin and melagatran should be further investigated clinically.

This study had some limitations. First, this study was based on the gene expression profiles of PBMs, which are not equal to those of osteoclasts. Second, in vivo and in vitro studies are warranted to dissect the functions of these biomarkers. Third, the use of biomarkers for diagnosis and treatment requires long-term clinical studies.

## Conclusion

In conclusion, the data in the present study showed that *BRSK2* and *VPS35* may be important diagnostic biomarkers for OP. Moreover, hesperidin and melagatran may be valuable OP treatment drugs that target *BRSK2* and *VPS35*. However, further studies are required to elucidate the relationship between these genes and OP.

### Electronic supplementary material

Below is the link to the electronic supplementary material.

Supplementary Material 1

Supplementary Material 2

Supplementary Material 3

Supplementary Material 4

Supplementary Material 5

## Data Availability

The data that support the findings of this study are available on request from the corresponding author.

## Abbreviations

OP: Osteoporosis
PCDs: Programmed cell death-related genes
DEGs: differentially expressed genes
BP: biological process
MF: molecular functions
CC: cellular components
PPI: Protein-protein interaction
SVMs: Support Vector Machines
RFE: Recursive Feature Elimination
RF: Random forest
ROC: receiver operating characteristic
GSEA: Gene Set Enrichment Analysis
TF: transcription factor
