# Supplementary figures and images for: Identification of potential cell death-related biomarkers for diagnosis and treatment of osteoporosis
Source: BMC Musculoskelet Disord. 2024 Mar 25;25:235. doi: 10.1186/s12891-024-07349-6 (PMC10964579; doi:10.1186/s12891-024-07349-6)

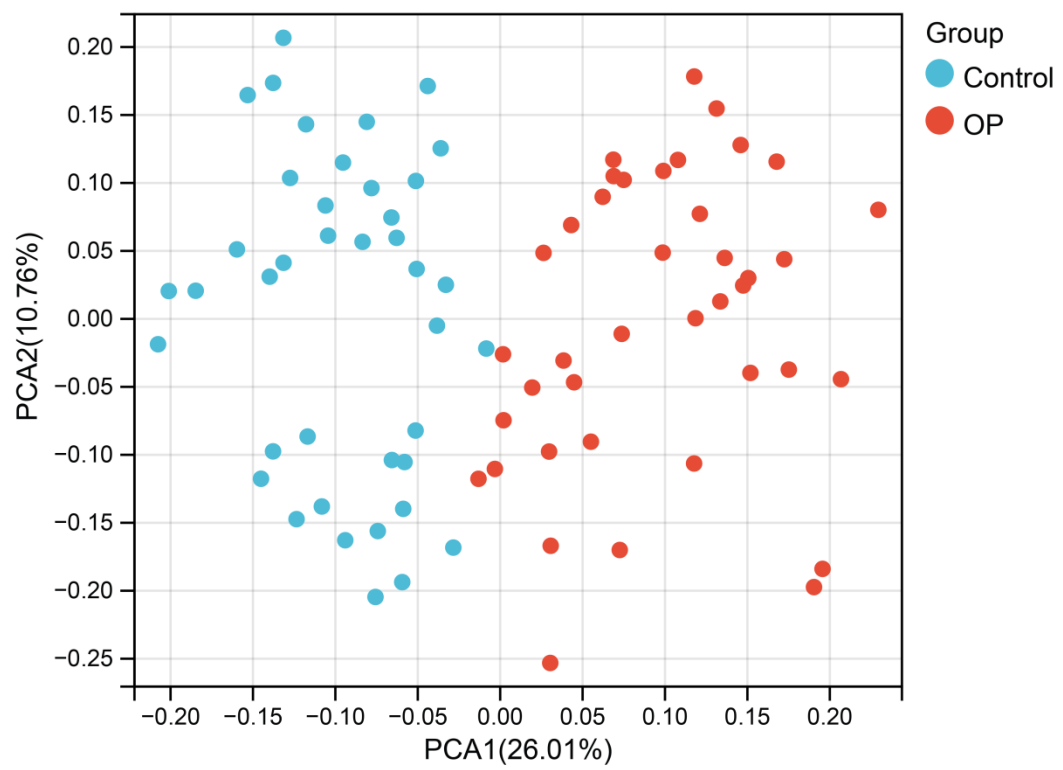

**Supplementary Figure 1** Principle component analysis of the samples at gene expression level.

Supplement: Supplementary file 4 — Supplementary Material 4 [file 12891_2024_7349_MOESM4_ESM.pdf]

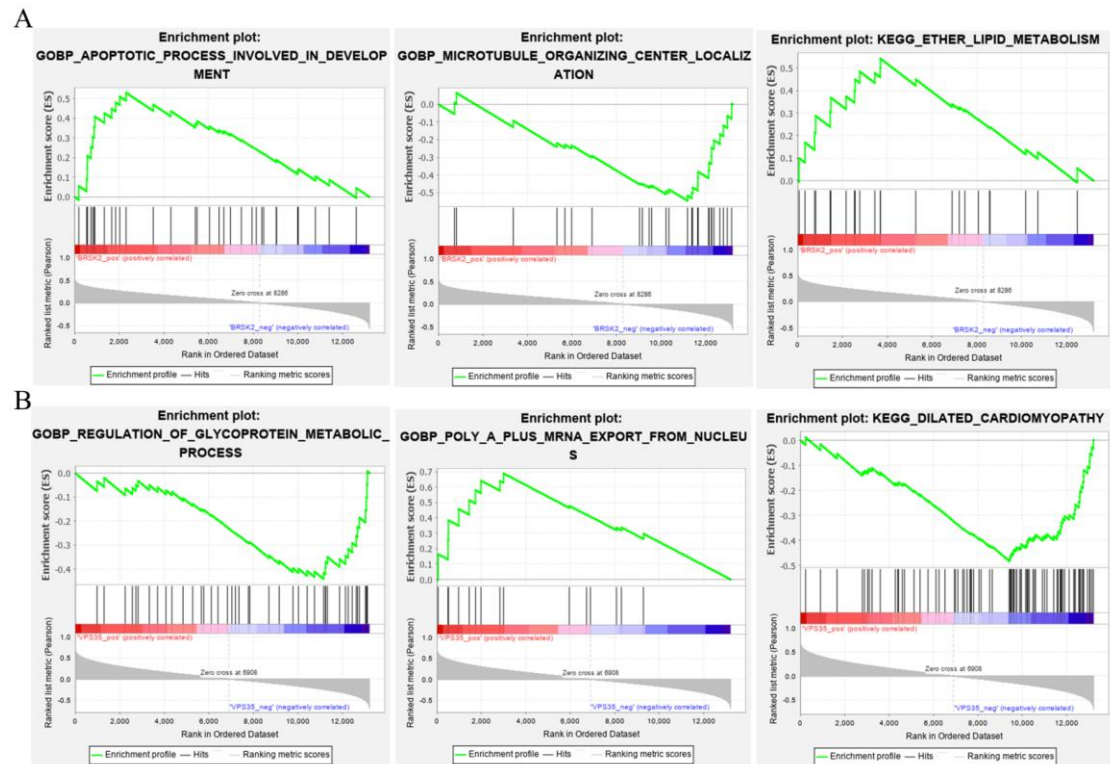

**Supplementary Figure 2** Functional enrichment analysis of BRSK2 and VPS35 in OP.

Supplement: Supplementary file 5 — Supplementary Material 5 [file 12891_2024_7349_MOESM5_ESM.pdf]
